# Experimental Study on Fatigue Performance of Steel Used in U75V Rails

**DOI:** 10.3390/ma18204706

**Published:** 2025-10-14

**Authors:** Dan Xu, Guoxiong Liu, Xianfeng Wang, Hui Liu

**Affiliations:** 1 China Railway 11th Bureau Group Corporation Limited, Wuhan 430064, China; xudan.11g@crcc.cn (D.X.); zt11jlgx@163.com (G.L.); 2 School of Civil Engineering and Architecture, Wuhan University of Technology, Wuhan 430070, China; 3 Sanya Science and Education Innovation Park, Wuhan University of Technology, Sanya 572025, China

**Keywords:** U75V rail, high-speed rail, monotonic tensile test, fatigue test, crack propagation test

## Abstract

The 60 kg/m U75V rail serves as the predominant rail type within China’s high-speed rail network. This study comprehensively evaluates the fatigue behavior of U75V rails through experimental investigations encompassing monotonic tensile testing, high-cycle fatigue characterization, and fatigue crack propagation analysis. All specimens were extracted from standardized 60 kg/m high-speed rail sections to ensure material consistency. Firstly, monotonic tensile tests were conducted to determine the fundamental mechanical properties of the U75V rail. Secondly, uniaxial tension–compression fatigue tests were conducted to establish the S-N and P-S-N relationships of the U75V rail. Lastly, fatigue crack propagation analysis was carried out on three compact tension specimens under three incremental loading forces. Monotonic tensile test results demonstrated full compliance of the material’s basic mechanical properties with Chinese national standards. Fatigue crack propagation results indicated that the crack growth rate of the U75V rail was not only related to the stress-intensity range ∆*K* but was also correlated with the loading force range ∆*F* due to a typical crack tip shielding effect, i.e., plasticity-induced crack closure effect. The derived fatigue performance parameters and crack growth mechanism provide essential inputs for predictive fatigue life modeling of high-speed rail infrastructure and development of refined finite element models for fatigue analysis.

## 1. Introduction

Steel rails constitute the backbone of modern railway systems, enabling efficient and safe transportation through optimized wheel–rail interactions under increasingly demanding operational conditions [1,2,3]. The global expansion of high-speed rail networks underscores the critical role of advanced rail steels in sustaining infrastructure longevity and operational reliability [4,5]. Rail degradation mechanisms, particularly rolling contact fatigue (RCF) and wear, remain primary challenges due to cyclic Hertzian stresses, plastic strain accumulation, and environmental factors [6,7,8,9]. These mechanisms are intrinsically linked to the metallurgical design of rail steels, where microstructure–property relationships govern performance under repetitive loading [10,11,12].

Historically, rail steels have evolved from wrought iron and early pearlitic alloys to modern hypereutectoid grades optimized for hardness and fatigue resistance [4]. The transition to fully pearlitic microstructures, refined through controlled cooling and alloying, has significantly enhanced wear resistance by reducing interlamellar spacing—a key determinant of strength and ductility [13,14,15]. For instance, heat-treated rails like U75V (Fe-0.75C-0.85Mn-0.6Si-0.084V wt%) achieve hardness values exceeding 370 HV through accelerated cooling, which suppresses proeutectoid cementite formation and refines pearlite colonies [16,17]. Such advancements align with the demands of high-speed networks, where rails must withstand large axle loads exceeding 22.5 tons and extreme contact pressures surpassing 1500 MPa [18,19,20,21].

Despite these innovations, RCF-induced crack initiation and propagation persist as critical failure modes, particularly in curved tracks and welded joints [8,9]. Fatigue cracks often originate at surface asperities or subsurface inclusions, propagating along shear bands formed by ratcheting deformation. The interplay between wear and fatigue further complicates rail longevity; while moderate wear removes surface cracks, excessive material loss compromises rail profile and accelerates RCF [22,23,24,25,26,27]. Recent studies emphasize the role of welding-induced residual stresses [28,29,30,31] and microstructure homogeneity [32,33] in dictating fatigue life. However, experimental data on fatigue crack growth kinetics and S-N behavior for specific rail grades, such as China’s U75V, remain scarce, limiting predictive modeling and maintenance optimization.

From October 2003, when the first 200 km/h high-speed railway was officially put into operation, to December 2024, China’s high-speed rail network has expended to an operating mileage of 48,000 km, developing into the world’s largest high-speed rail system within a span of just over two decades [34]. Within China’s high-speed rail network, the 60 kg/m U75V rail serves as the predominant rail type. Since the rails are continuously subjected to cyclic loading during operation, investigating the fatigue performance of U75V rails is critical for ensuring operational safety and optimizing maintenance strategies in the world’s largest high-speed railway system.

In this study, the fatigue behavior of U75V rails is comprehensively evaluated through experimental investigations encompassing monotonic tensile testing, high-cycle fatigue characterization, and fatigue crack propagation analysis. The derived fatigue performance parameters and crack growth mechanism provide essential inputs for predictive fatigue life modeling of high-speed rail infrastructure and development of refined finite element models for fatigue analysis. In Section 2, monotonic tensile testing is conducted to determine the fundamental mechanical properties of the employed rail segments in order to guarantee full compliance of the material’s basic mechanical properties with Chinese national standards for U75V rails. In Section 3, uniaxial tension–compression fatigue tests are conducted with a high-frequency fatigue testing system to reveal the S-N and P-S-N relationships of the U75V rails. In Section 4, fatigue crack propagation tests are conducted upon three compact tension specimens under three different loading conditions to provide proportional and exponential constants for the Paris–Erdogan equation and evaluate the crack growth mechanism of U75V rails. Section 5 concludes the study. It should be noted that Section 2, Section 3 and Section 4 contain independent methodology, results, and discussion regarding each of these three different types of experiment.

## 2. Basic Mechanical Properties of U75V Rails via Monotonic Tensile Testing

As the predominant rail type within China’s high-speed rail network, the chemical composition of U75V rail steel is presented in Table 1.

Monotonic tensile tests were first conducted to determine the fundamental mechanical properties of the standardized 60 kg/m U75V rail segment from which specimens were extracted. This procedure was implemented to verify compliance with Chinese national standards for material quality in the employed rail segments.

**Figure 1:**
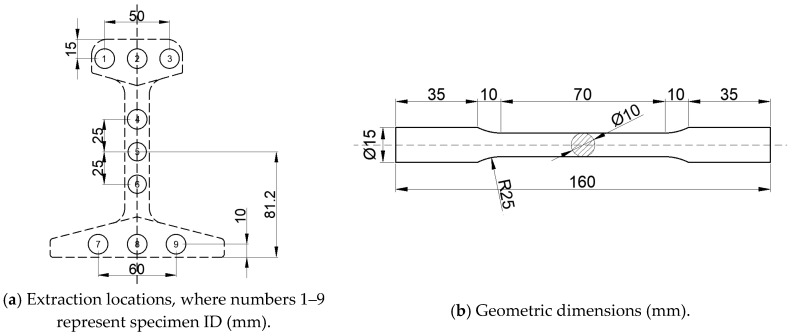
The extraction locations and geometric dimensions of the monotonic tensile specimens.

The test methodology and machining dimensions were determined in accordance with Chinese national standard GB/T 228.1-2021 Metallic materials-Tensile testing-Part 1: Method of test at room temperature [36]. In the monotonic tensile test, three specimens each were extracted from the rail head, rail web, and rail base (totaling nine specimens), with their extraction locations and geometric dimensions detailed in Figure 1.

**Figure 2:**
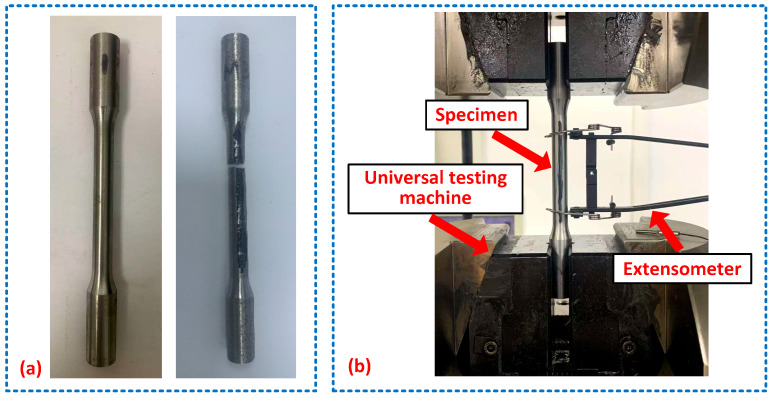
Experimental setup of the monotonic tensile test: (**a**) monotonic tensile specimens before and after failure; (**b**) monotonic tensile test system.

The experimental setup of the monotonic tensile test is presented in Figure 2. The monotonic tensile tests were conducted on a universal testing machine at a tensile rate of 5 mm/min. During the tensile process, load and strain were recorded by the automatic data acquisition system integrated into the loading system and an extensometer, respectively, as presented in Figure 2.

**Figure 3:**
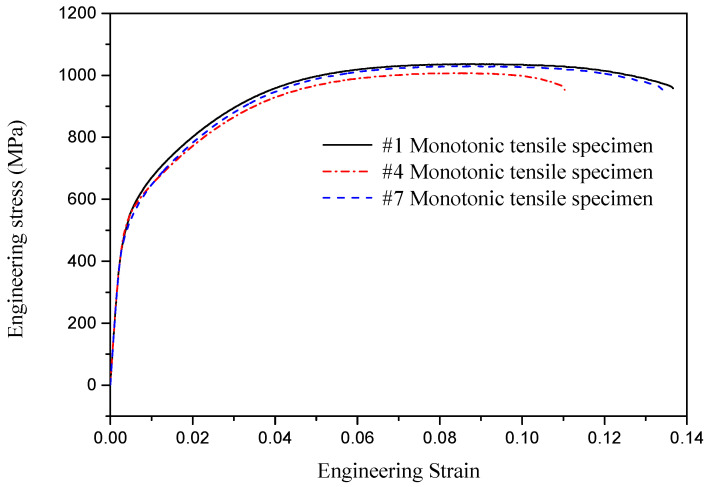
Engineering stress–strain curves of different specimens.

As revealed in Figure 2a, the fractured specimen exhibits negligible necking at the fracture site, a relatively flat fracture surface morphology, and low elongation after fracture, collectively manifesting the characteristic fracture behavior of high-strength steels. The basic mechanical properties of the nine specimens derived from experimental data are summarized in Table 2. In order to show the characteristics of the stress–strain relationship of U75V rail steel, typical engineering stress–strain curves for three of the nine specimens from the rail head, rail web, and rail base, i.e., specimens #1, #4, and #7, are presented in Figure 3. It should be noted that U75V rail steel is a high-strength steel that exhibits no distinct yield plateau during tensile deformation, as shown in Figure 3, and that the yield strength is represented by 0.2% offset yield strength (*Rp*0.2). As specified in China’s standard for hot-rolled steel rails for railways [35], U75V rails must exhibit a tensile strength of ≥980 MPa and an elongation after fracture of ≥10%. The tabulated results demonstrate that all tested specimens exceed the standard-mandated thresholds, confirming compliance with regulatory requirements. This validates that the mechanical properties of the employed rail segments meet the prescribed criteria.

## 3. Fatigue Characterization of U75v Rails via Uniaxial Tension–Compression Fatigue Testing

Secondly, high-cycle uniaxial tension–compression fatigue tests were conducted to characterize the fatigue performance of U75V rails, i.e., establishing the S-N and P-S-N relationships of U75V rails.

**Figure 4:**
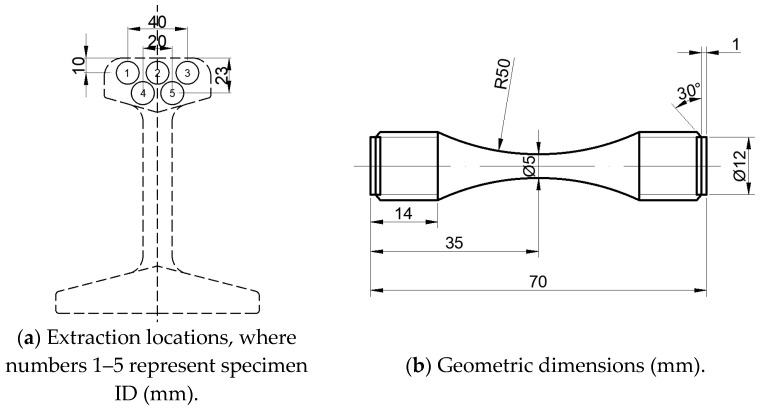
The extraction locations and geometric dimensions of the fatigue test specimens in the high-frequency uniaxial tension–compression fatigue test.

Given that the rail head directly sustains train loading, specimens for high-cycle fatigue testing were extracted from this critical region. Five specimens each were obtained from three rail heads, yielding a total of fifteen specimens for experimental evaluation, with their extraction locations detailed in Figure 4a. The test methodology and machining dimensions were determined in accordance with Chinese national standard GB/T 3075-2021 Metallic Materials-Fatigue Testing-Axial force-controlled method [37]. The detailed geometric dimensions of the specimens are specified in Figure 4b.

**Figure 5:**
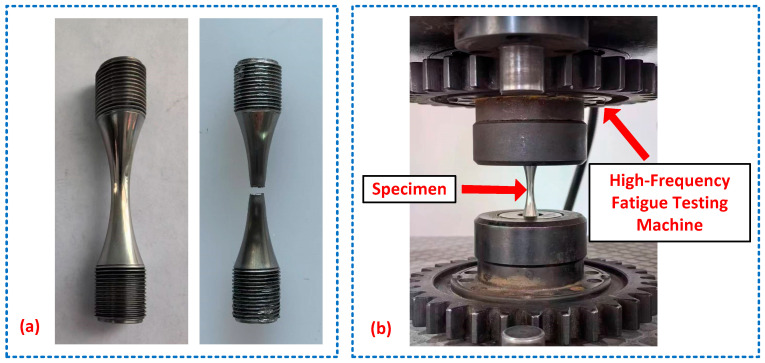
Experimental setup of the high-cycle fatigue test: (**a**) fatigue test specimens before and after failure; (**b**) high-cycle fatigue test system.

The experimental setup of the high-cycle fatigue test is presented in Figure 5. The uniaxial tension–compression fatigue tests were conducted using a high-frequency fatigue testing machine with a loading frequency of 140 Hz and a stress ratio of −1. Five stress amplitudes were selected, dropping from 610 MPa to 510 MPa with 25 MPa gapping, and three sets of replicates were performed for each stress amplitude. Note that the largest stress amplitude was selected using 60% of the tensile strength of the U75V rail steel obtained in Section 2. The tests were terminated when either specimen fracture occurred, or the predetermined life limit of 5 million cycles was reached. During testing, specimen temperature was continuously monitored, and the tests were paused if the temperature rise exceeded 50 °C.

Figure 5a illustrates the failure characteristics of the fatigue specimens, revealing flat fracture surfaces with no significant necking observed, consistent with the fatigue fracture behavior of typical high-strength, low-ductility steels. The uniaxial tension–compression fatigue test data for the U75V rails are summarized in Table 3, which documents the applied stress amplitudes $\sigma_{a}$, loading frequencies, and corresponding fatigue life $N_{f}$.

**Figure 6:**
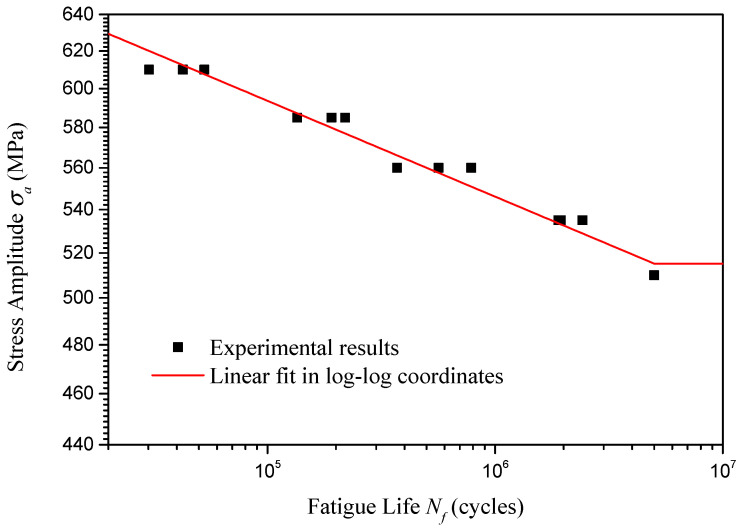
Basic S-N curve of U75V rails.

Based on the fatigue test data, using linear fitting in log–log coordinates through the least squares fitting method, Figure 6 presents the basic S-N curve (when stress ratio is −1) for the U75V rails in the high-cycle fatigue scenario. The expression of the basic S-N curve is shown in the following equation

(1) $$\mathrm{lg}\left(\sigma_{a}\right)=-0.03627\mathrm{lg}\left(N_{f}\right)+2.955$$

and the adjusted coefficient of determination $R_{adjust}^{2}=0.9743$. Substituting $N_{f}=5\times 10^{6}$ into Equation (1), the high-cycle fatigue strength of U75V rails is revealed to be 515 MPa.

For a given survival rate *P*, the logarithmic value of the probabilistic fatigue life $x_{P}$ could be expressed as

(2) $$x_{P}=x_{mean}+U_{P}\beta x_{stdev}$$

where $x_{mean}$ is the mean of the $lg\left(N_{f}\right)$ and $x_{stdev}$ is the standard deviation of $lg\left(N_{f}\right)$ for a certain $lg\left(\sigma_{a}\right)$. Herein, $N_{f}$ represents the fatigue test data. β is the correction coefficient of standard deviation, for which we take 1.128 for the three samples. $U_{P}$ is the standard normal deviation, for which we take 0, −1.282, and −3.09, respectively, for a 50%, 90%, and 99.9% survival rate. Combining the fatigue test data presented in Table 3 and Equation (2), the calculated logarithmic results of the probabilistic fatigue life are summarized in Table 4.

**Figure 7:**
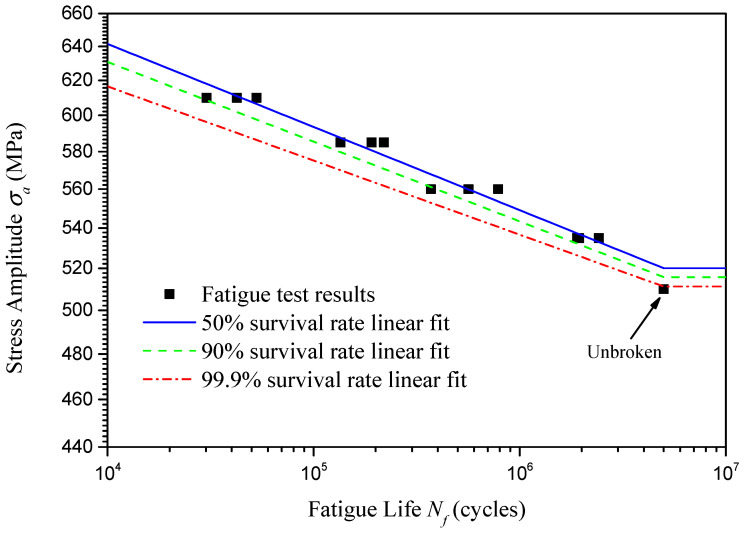
P-S-N curve of U75V rails.

Based on the probabilistic fatigue life results presented in Table 4, using linear fitting of $lg\left(\sigma_{a}\right)$ and $lg\left(N_{f}\right)$ for different survival rates through the least squares fitting method, Figure 7 presents the P-S-N curves for the U75V rails. The expressions of the P-S-N curves for different survival rates are shown in the following equations

(3) $$\begin{array}{l} P=50\%:\mathrm{lg}\left(\sigma_{a}\right)=-0.03377\mathrm{lg}\left(N_{f}\right)+2.942\\ P=90\%:\mathrm{lg}\left(\sigma_{a}\right)=-0.03239\mathrm{lg}\left(N_{f}\right)+2.929\\ P=99.9\%:\mathrm{lg}\left(\sigma_{a}\right)=-0.03012\mathrm{lg}\left(N_{f}\right)+2.910 \end{array}$$

And the adjusted coefficients of determination $R_{adjust}^{2}$ are 0.9943, 0.9855, and 0.9500, respectively, for the linear fitting of a 50%, 90%, and 99.9% survival rate.

Theoretically, as fatigue life increases, the P-S-N curve should exhibit an expanding shape. However, in Figure 7, the P-S-N curve shows a contracting shape as fatigue life increases. This is because the number of specimens is small, and insufficient sampling results in the standard deviation of fatigue life corresponding to 535 MPa being much smaller than the standard deviation of fatigue life corresponding to other stress amplitudes. If the number of specimens were sufficient, the standard deviation of fatigue life would increase as the stress amplitude decreased.

## 4. Fatigue Crack Propagation Test of U75v Rails

Lastly, fatigue crack propagation tests were carried out on the compact tension (CT) specimen to reveal the crack growth kinetics of the U75V rails.

**Figure 8:**
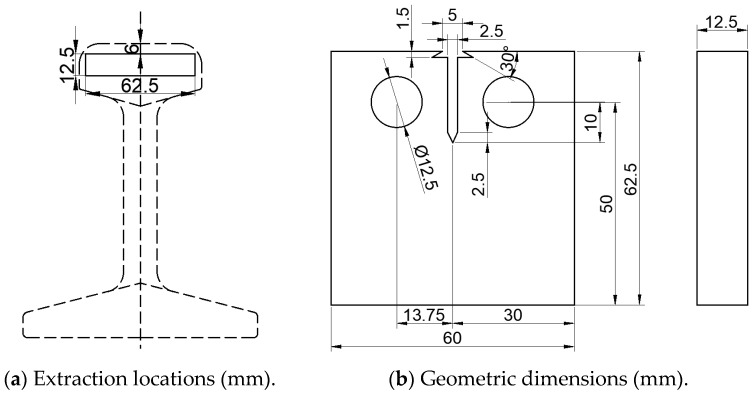
The extraction locations and geometric dimensions of the compact tension specimens in crack propagation test.

Similarly to the sampling location of the fatigue test, three compact tension specimens for the crack propagation test were extracted from three rail heads, with their extraction locations detailed in Figure 8a. The test methodology and machining dimensions were determined in accordance with Chinese national standard GB/T 6398-2017 Metallic Materials-Fatigue Testing-Fatigue crack growth method [38]. The detailed geometric dimensions of the CT specimens are specified in Figure 8b.

**Figure 9:**
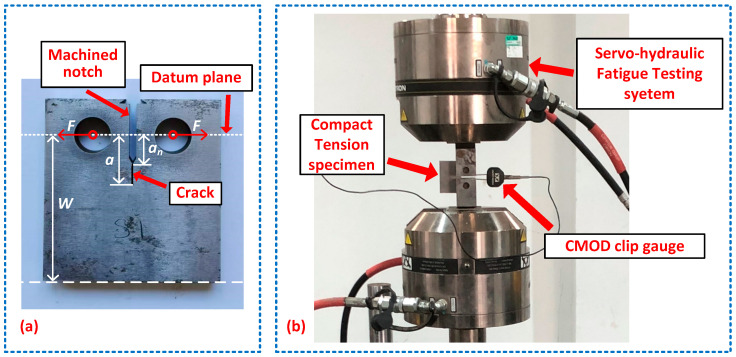
Experimental setup of the crack propagation test: (**a**) compact Tension specimens; (**b**) crack propagation test system.

The experimental setup of the fatigue crack propagation test is presented in Figure 9. The fatigue crack propagation tests on three CT specimens were conducted using an Instron 8801 servo-hydraulic fatigue testing system. The loading cycles *N* and the opening displacement of the machined notch were recorded, respectively, by the fatigue testing system and a crack mouth opening displacement (CMOD) clip gauge. The fatigue crack length *a* is defined as the distance from the datum plane to the crack tip as illustrated in Figure 9a. It should be noted that the crack length *a* is not directly measured but converted from the crack opening displacement using the compliance method.

Before the fatigue crack propagation test began, an initial crack with a length of $a_{p}=2\;mm$ was prefabricated using a decreasing *K* method. And then, the crack propagation test was conducted using an increasing *K* method, where *K* is the stress-intensity factor defined as

(4) $$K=\frac{F}{B\sqrt{W}}g\left(\frac{a}{W}\right)$$

where *F* is the applied force, *B* is the specimen thickness, *W* is the specimen width as illustrated in Figure 9a, and *g*(*a*/*W*) is the form factor of CT specimens expressed as

(5) $$g\left(\frac{a}{W}\right)=\frac{\left(2+\frac{a}{W}\right)}{{\left(1-\frac{a}{W}\right)}^{\frac{3}{2}}}\left[0.886+4.64\frac{a}{W}-13.32{\left(\frac{a}{W}\right)}^{2}+14.72{\left(\frac{a}{W}\right)}^{3}-5.6{\left(\frac{a}{W}\right)}^{4}\right]$$

where $0.2\leq \left(a/W\right)\leq 0.8$.

In the crack prefabrication and the crack propagation test, harmonic loading was applied, where the maximum and minimum force in one loading cycle was defined as $F_{max}$ and $F_{min}$. Respectively substituting $F_{max}$ and $F_{min}$ into Equation (4), the maximum and minimum stress-intensity factors $K_{max}$ and $K_{min}$ could be obtained. In addition, the stress ratio during the crack propagation process was defined as $F_{min}/F_{max}$.

In the crack prefabrication process, the loading frequency and the stress ratio were constant, while $K_{max}$ decreased from an initial value to a completion value until the prefabricated crack length reached the target value of $a_{p}=2\;mm$. The crack prefabrication settings, including loading frequency, stress ratio, and maximum stress-intensity factor $K_{max}$ at the start and completion of crack prefabrication, are summarized in Table 5.

In the crack propagation test, the loading frequency, the stress ratio, and the maximum applied force $F_{max}$ were constant. During the loading process, the crack length *a* continued to increase, leading to an increasing $K_{max}$. The beginning $K_{max}$ during the crack propagation test was consistent with the end $K_{max}$ in the crack prefabrication, as listed in Table 5. Therefore, the applied force $F_{max}$ in the crack propagation could be determined by substituting $a=a_{n}+a_{p}=12\;mm$ and the end $K_{max}$ listed in Table 5 into Equation (4), where $a_{n}=10\;mm$ is the length of the machined notch as illustrated in Figure 9a. The crack propagation settings including loading frequency, stress ratio, and the applied force $F_{max},$ are summarized in Table 6.

**Figure 10:**
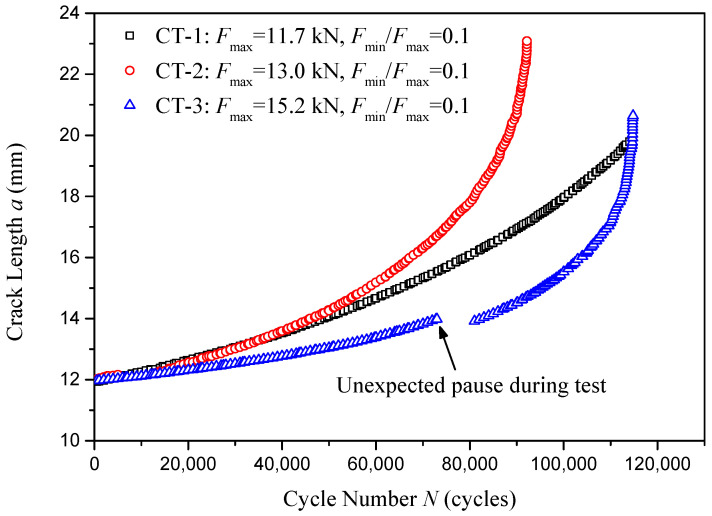
Crack length *a* versus loading cycles *N* in crack propagation test.

Three compact tension specimens extracted from standard 60 kg/m U75V rails were cyclically loaded under three different applied forces $F_{max}$, i.e., 11.7 kN, 13.0 kN, and 15.2 kN. For each loading force, the crack length *a* with respect to the loading cycles *N* is presented in Figure 10. It should be noted that an unexpected pause occurred during the testing of specimen CT-3 at 7300 loading cycles. The loading system soon restarted, and the *a*-*N* data recording was restarted at 81,000 loading cycles. Normally, loading pauses should be avoided during crack propagation testing, since it will cause data discontinuities, as shown in Figure 10. In the following data processing regarding crack growth analysis, the *a*-*N* data from CT-3 is processed as two separate pieces.

Fatigue crack growth in materials under cyclic loading typically occurs in three distinct stages, i.e., Stage I crack initiation, Stage II stable crack propagation, and Stage III unstable fracture. This study focuses on the stable crack propagation stage, when the crack grows in a direction perpendicular to the applied tensile stress, following a stable, predictable path. In the stable crack propagation stage, the crack growth rate *da*/*dN* correlates with the stress-intensity factor range ∆*K* ($∆K=K_{max}-K_{min}$) via empirical laws, among which the Paris–Erdogan equation is the most commonly used, expressed as [39].

(6) $$\frac{da}{dN}=C{\left(\Delta K\right)}^{m}$$

where *C* and *m* are, respectively, the proportional constant and exponential constant determined through experimentation.

To determine the proportional and exponential constants *C* and *m* of the U75V rails, the correlation between the crack growth rate *da*/*dN* and the stress-intensity factor range ∆*K* based on the experimental data needs to be clarified. The crack growth rate *da*/*dN* could be derived from the experimentally obtained *a*-*N* curve presented in Figure 10. Specifically, local polynomial fitting using the seven-point incremental method was performed on the *a*-*N* curve, followed by subsequent differentiation to determine the fitted crack length $\hat{a}$ and corresponding crack growth rate $d\hat{a}/dN$. For the *i*-th crack length data point $a_{i}$, a seven-point dataset comprising three preceding and three succeeding data points centered at $a_{i}$ was selected. A second-order polynomial fitting was applied to these seven data points, allowing the fitted crack length ${\hat{a}}_{i}$ to be expressed as

(7) $${\hat{a}}_{i}=b_{0}+b_{1}\left(\frac{N_{i}-C_{1}}{C_{2}}\right)+b_{2}{\left(\frac{N_{i}-C_{1}}{C_{2}}\right)}^{2}$$

where $N_{i}$ is the loading cycle number corresponding to $a_{i}$, and *i* ≥ 4. $b_{0}$, $b_{1},$ and $b_{2}$ are the regression parameters determined by the least squares method. $C_{1}$ and $C_{2}$ are the parameters used to transform the input data expressed as

(8) $$\begin{array}{l} C_{1}=\frac{1}{2}\left(N_{i-3}+N_{i+3}\right)\\ C_{2}=\frac{1}{2}\left(N_{i+3}-N_{i-3}\right) \end{array}$$

And the *i*-th crack growth rate ${\left(da/dN\right)}_{i}$ can be determined by differentiating the fitted crack length ${\hat{a}}_{i}$ with respect to the number of load cycles $N_{i}$ as

(9) $${\left(\frac{da}{dN}\right)}_{i}=\frac{b_{1}}{C_{2}}+2b_{2}\left(\frac{N_{i}-C_{1}}{C_{2}}\right)$$

As for the *i*-th stress-intensity factor range ${∆K}_{i}$, since the crack propagation test is conducted under a constant force condition, ${∆K}_{i}$ could be expressed with respect to the actual crack length $a_{i}$ using Equation (4) as

(10) $$\Delta K_{i}=\frac{\Delta F}{B\sqrt{W}}g\left(\frac{a_{i}}{W}\right)$$

where $∆F=F_{max}-F_{min}$ is the applied force range.

**Figure 11:**
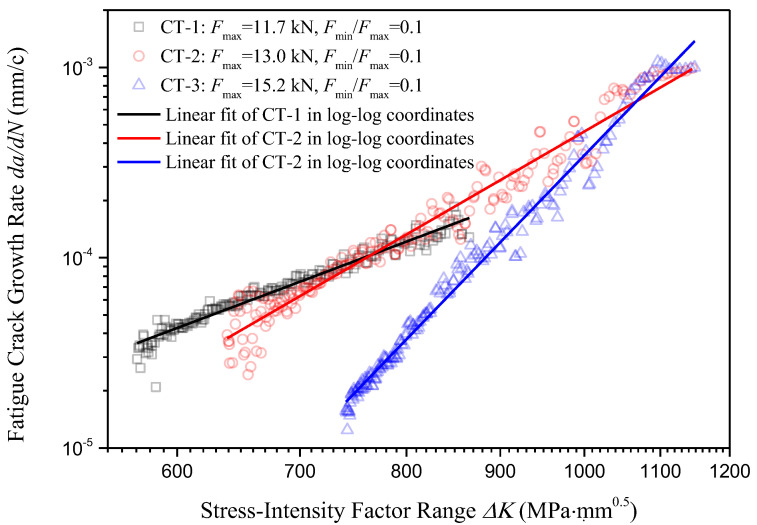
Fatigue crack growth rate *da*/*dN* with respect to stress-intensity factor range ∆*K*.

The scatters of the *i*-th crack growth rate ${\left(da/dN\right)}_{i}$ with respect to the *i*-th stress-intensity factor range ${∆K}_{i}$ under three different loading forces are presented in a log–log scale as shown in Figure 11. Note that the units of ∆*K* in Figure 11 are MPa∙mm^0.5^ instead of MPa∙m^0.5^, which were employed in Table 6.

The commonly used Paris–Erdogan equation describing the crack propagation kinetics could be rewritten in log–log scale as

(11) $$\mathrm{lg}\left(\frac{da}{dN}\right)=m\mathrm{lg}\left(\Delta K\right)+\mathrm{lg}C$$

Which presents a linear correlation between the crack growth rate *da*/*dN* and the stress-intensity factor range ∆*K* in the log–log scale. Based on the processed crack propagation data presented in Figure 11, using linear fitting in log–log coordinates through the least squares fitting method, the crack growth rate with respect to the stress-intensity factor range for three different loading cases could be expressed as

(12) $$\begin{array}{l} \mathrm{CT}1:\mathrm{lg}\left(\frac{da}{dN}\right)=3.6347\mathrm{lg}\left(\Delta K\right)-14.4672\\ \mathrm{CT}2:\mathrm{lg}\left(\frac{da}{dN}\right)=5.5842\mathrm{lg}\left(\Delta K\right)-20.0893\\ \mathrm{CT}3:\mathrm{lg}\left(\frac{da}{dN}\right)=9.9805\mathrm{lg}\left(\Delta K\right)-33.4033 \end{array}$$

Equation (12) could be rewritten in the original form of Paris–Erdogan equation as

(13) $$\begin{array}{l} \mathrm{CT}1:\frac{da}{dN}=3.41\times 10^{-15}{\left(\Delta K\right)}^{3.6347}\\ \mathrm{CT}2:\frac{da}{dN}=8.14\times 10^{-21}{\left(\Delta K\right)}^{5.5842}\\ \mathrm{CT}3:\frac{da}{dN}=3.95\times 10^{-34}{\left(\Delta K\right)}^{9.9805} \end{array}$$

The experimentally determined proportional and exponential constants for the Paris–Erdogan equation under different loading conditions are summarized in Table 7 and compared with a recently published investigation [40]. In Ma et al.’s study, crack propagation tests on two CT specimens were conducted under two different uniformly distributed loading forces, and the crack initiation site identification as well as microstructure analysis of U75V rails are included. It was noticed that the crack propagation pattern was affected by applied load, but it was not further discussed in Ma et al.’s study. Ma et al. provided the experimentally obtained crack propagation formula for U75V rail steel under the lower applied load, i.e., $da/dN=2.3157{\;\times \;10^{-9}\left(\Delta K\right)}^{3.2359}$, where the unit of da/dN is millimeter and the unit of ΔK is $MPa\sqrt{m}$. In order to unify the unit with this study, substituting $\left(\Delta K\right)/\sqrt{1000}$ into ΔK, Ma et al.’s result is transferred to $da/dN=3.2422{\;\times \;10^{-14}\left(\Delta K\right)}^{3.2359}$. The experimentally obtained *C* and *m* by Ma et al. are provided in Table 7 for comparison. As we can see in Table 7, Ma et al.’s results are very close to the present results obtained upon specimen CT-1.

Combining Figure 11 and Table 7, it is observed that for each of these three loading forces, the Paris’ law is well performed, and the adjusted coefficients of determination $R_{adjust}^{2}$ for all linear regressions in log–log scale are over 0.95. However, different loading forces yield different constants for the Paris–Erdogan equation, i.e., when the loading force $F_{max}$ increases from 11.7 kN to 15.2 kN, the constant *m* increases from 3.6347 to 9.9805, and the constant *C* decreases from 3.41 × 10^−15^ to 3.95 × 10^−34^. This phenomenon indicates that the crack growth rate for U75V rails is not only related to the stress-intensity range ∆*K* (which represents the stress field intensity at the crack tip), but also correlated with the loading force range ∆*F* (correlated with the far-field stress intensity of the crack tip).

To explain this phenomenon, we need to look into Figure 11. At the beginning of crack propagation, for these three different loading cases, higher stress field intensity at the crack tip yields a similar or even lower crack propagation rate, which means the effective driving force for crack propagation has been further reduced due to a higher loading force. The mechanical process that reduces the effective driving force acting on the crack tip is called the crack tip shielding effect [39]. Considering these three loading cases have the same initial crack length, the crack tip shielding effect acting in the present phenomenon would be the plasticity-induced crack closure effect [41]. Specifically, a plastic zone forms at the crack tip under high $K_{max}$. During unloading, the surrounding elastic material tries to return to its original shape, but the residual tensile deformation in the plastic zone “presses against” the crack surface, causing the crack to engage prematurely during the initial stage of loading. The above analysis implies that the influence of the plasticity-induced crack closure effect on the engineering fatigue performance of U75V rail steel could be expressed through variations in the constants *C* and *m*.

Traditionally, the proportional and exponential constants *C* and *m* are considered to be material constants, which means that for the same material under different loading conditions, the experimentally determined *C* and *m* should stay the same, or at least close to each other. However, the present experimental results show that the constants *C* and *m* for U75V rail steel are monotonously affected by the loading force range ∆*F* due to the plasticity-induced crack closure effect, which indicates that *C* and *m* are not absolute material constants but more like calculated constants correlated with the average stress intensity in the specimen.

In the Paris–Erdogan equation, the exponential constant *m* is a sensitivity indicator reflecting the sensitivity of the crack propagation rate to ∆*K*, and the proportional constant *C* is an absolute rate scale determining the absolute crack propagation rate under the same ∆*K*. For U75V rails, reviewing the experimentally obtained monotonic correlations between ∆*F* and the constants *C* and *m*, it is found that, in a low working-stress environment, even a low stress-intensity factor range ∆*K* could yield considerable crack propagation rate, and in a high working-stress environment, the crack propagation rate is low at first but extremely sensitive to ∆*K,* leading to a rapid propagation rate at the last stage of crack propagation. As for the enlightenment of the experimental conclusions on rail maintenance strategies, for light-duty passenger railways, the maintenance strategy should favor the early detection and elimination of cracks; for heavy-duty freight railways, the crack detection cycle should be shortened in the middle and late stages of rail service.

Moreover, if we look into the form of the Paris–Erdogan equation, where *C* and *m* are traditionally considered to be material constants, the driving force of crack propagation comes only from the stress-intensity factor range ∆*K*, which is the quantified expression of the stress field intensity at the crack tip. In the present study, *C* and *m* are found not to be absolute material constants but correlated with the average stress intensity in the specimen due to the plasticity-induced crack closure effect. Which means that the crack growth kinetics are not only related to the stress field intensity at the crack tip but also correlated with the working stress intensity in engineering practice. As for quantitative characterization of the correlation between the crack propagation rate and the applied nominal stress, substantial tests are required in future studies.

## 5. Conclusions

In the present study, the fatigue performance of U75V rails is experimentally evaluated, encompassing monotonic tensile testing, high-cycle fatigue behavior characterization, and fatigue crack propagation analysis. All specimens employed in this study were extracted from standardized 60 kg/m high-speed rail sections.

In the monotonic tensile test, the fundamental mechanical properties of the employed rail segments were determined, including elastic modulus, tensile strength, yield strength, and elongation after fracture. The experimental results demonstrated full compliance of the material’s basic mechanical properties with Chinese national standards for U75V high-speed rails.

In the high-cycle fatigue behavior characterization, uniaxial tension–compression fatigue tests were conducted with a high-frequency fatigue testing system with a loading frequency of 140 Hz and a stress ratio of −1. Five stress amplitudes were selected, dropping from 610 MPa to 510MPa with 25 MPa gapping, and three sets of replicates were performed for each stress amplitude. Using the experimental results, the basic S-N relationship of U75V rails was revealed to be $lg\left(\sigma_{a}\right)=-0.03627lg\left(N_{f}\right)+2.955$, and a high-cycle fatigue strength at 5 million cycles was obtained at 515 MPa. For different survival rates *P*, the P-S-N curves and corresponding expressions of U75V rails are also provided in the present study.

In the fatigue crack propagation tests, three CT specimens were tested with a loading frequency of 10 Hz and a stress ratio of 0.1. The crack propagation tests were carried out under three different maximum loading forces, i.e., 11.7 kN, 13.0 kN, and 15.2 kN. It was observed that for each of these three loading forces, Paris’ law was well performed, but different loading forces yield different constants for the Paris–Erdogan equation, i.e., when the loading force $F_{max}$ increases from 11.7 kN to 15.2 kN, the constant *m* increases from 3.6347 to 9.9805, and the constant *C* decreases from 3.41 × 10^−15^ to 3.95 × 10^−34^. The experimental results indicate that the crack growth rate for U75V rails is not only related to the stress-intensity range ∆*K* but also correlated with the loading force range ∆*F*, which means *C* and *m* are not absolute material constants but more like calculated constants correlated with the average stress in the specimen. Based on the experimental results, the authors suppose that this phenomenon is induced by a typical crack tip shielding effect, i.e., a plasticity-induced crack closure effect. Specifically, a plastic zone forms at the crack tip under high $K_{max}$. During unloading, the surrounding elastic material tries to return to its original shape, but the residual tensile deformation in the plastic zone “presses against” the crack surface, causing the inhibition of crack propagation. The present discovery implies that the influence of plasticity-induced crack closure effect on the engineering fatigue performance of U75V rail steel could be expressed through variations in the constants *C* and *m*.

## Figures and Tables

**Table 1:** Chemical composition of U75V rail steel (wt%) [35].

C	Mn	Si	V	P	S	Al_t_
0.71–0.80	0.70–1.05	0.50–0.80	0.04–0.12	≤0.025	≤0.025	≤0.004

**Table 2:** Basic mechanical properties of monotonic tensile specimens extracted from 60 kg/m U75V rails.

Specimen ID	Elastic Modulus *E* (GPa)	Tensile Strength *Rm* (MPa)	Yield Strength *Rp*0.2 (MPa)	Elongation After Fracture (%)
#1	195.08	1036.5	553.81	13.675
#2	198.13	1027.2	620	10.623
#3	197.19	1047.8	552.58	12.306
#4	199.19	1007	548.5	11.034
#5	196.68	985.6	529.6	14.427
#6	199.73	972.3	526.63	11.692
#7	196.62	1029.4	526.34	13.404
#8	197.23	1032.6	598.12	11.934
#9	191.53	1034.9	500.11	13.818
Average	196.82	1019.3	550.63	12.546
Standard Deviation	2.29	24.0	35.35	1.261
Coefficient of Variation	0.012	0.024	0.064	0.101

**Table 3:** High-cycle fatigue test results.

Stress Amplitude $\sigma_{a}$ (MPa)	Loading Frequency (Hz)	Fatigue Life $N_{f}$ (Cycles)
610	138	52,800
610	138	42,500
610	139	30,200
585	140	135,000
585	141	191,200
585	141	219,200
560	140	370,700
560	140	564,700
560	139	785,400
535	138	1,942,000
535	141	2,421,000
535	141	1,898,000
510	141	5 × 10^6^ Unbroken
510	140	5 × 10^6^ Unbroken
510	140	5 × 10^6^ Unbroken

**Table 4:** Probabilistic fatigue life results.

Stress Amplitude $\sigma_{a}$ (MPa)	$lg\left(\sigma_{a}\right)$	Mean of $lg\left(N_{f}\right)$	Standard Deviation of $lg\left(N_{f}\right)$	50% Survival Rate $lg\left(N_{f}\right)$	90% Survival Rate $lg\left(N_{f}\right)$	99.9% Survival Rate $lg\left(N_{f}\right)$
610	2.78533	4.61034	0.12232	4.61034	4.43346	4.18401
585	2.76716	5.25089	0.10854	5.25089	5.09393	4.87257
560	2.74819	5.73864	0.16343	5.73864	5.5023	5.169
535	2.72835	6.31685	0.05836	6.31685	6.23245	6.11342
510	2.70757	6.69897	0	Not applicable	Not applicable	Not applicable

**Table 5:** Crack prefabrication settings.

Specimen ID	Loading Frequency (Hz)	Stress Ratio	Start $K_{max}$ $(MPa\sqrt{m}$)	End $K_{max}$ $(MPa\sqrt{m}$)
CT-1	10	0.1	26.967	20
CT-2	10	0.1	29.678	22
CT-3	10	0.1	33.901	26

**Table 6:** Crack propagation test settings.

Specimen ID	Loading Frequency (Hz)	Stress Ratio	Applied Force $F_{max}$ (kN)	Start $K_{max}$ $(MPa\sqrt{m}$)
CT-1	10	0.1	11.7	20
CT-2	10	0.1	13.0	22
CT-3	10	0.1	15.2	26

**Table 7:** Experimentally determined constants for Paris’ law under different loading forces.

Specimen ID	Applied Force $F_{max}$ (kN)	Stress Ratio	Proportional Constant *C*	Exponential Constant *m*	Adjusted Coefficient of Determination $R_{adjust}^{2}$
CT-1	11.7	0.1	3.41 × 10^−15^	3.6347	0.9596
CT-2	13.0	0.1	8.14 × 10^−21^	5.5842	0.9600
CT-3	15.2	0.1	3.95 × 10^−34^	9.9805	0.9861
Ma et al. [40]	Uniformly distributed 13.0	0.1	3.24 × 10^−14^	3.2359	--

## Data Availability

The original contributions presented in this study are included in the article. Further inquiries can be directed to the corresponding authors.
